# Supplementary material for: The genome sequence of the Scorched Wing moth, Plagodis dolabraria (Linnaeus, 1767)
Source: Wellcome Open Res. 2025 Feb 28;10:111. [Version 1] doi: 10.12688/wellcomeopenres.23761.1 (PMC11926530; doi:10.12688/wellcomeopenres.23761.1)
Supplement: Peer review history

# Reviewer report: Reviewer response for version 1

**DOI:** 10.21956/wellcomeopenres.26209.r119964

**Published:** 2025-03-20

**Authors:** Maríndia Deprá

**Affiliations:** 1 Federal University of Rio Grande do Sul, Porto Alegre, Brazil

The data note titled “The genome sequence of the Scorched Wing moth, *Plagodis dolabraria* (Linnaeus, 1767)” presents a well-structured genome assembly from a male specimen of Plagodis dolabraria. The study generated nuclear and mitochondrial genome sequences using advanced technologies, ensuring robust chromosomal assembly and accurate gene annotation.

 The haploid genome size is 931.66 Mb, assigned to 31 chromosomal pseudomolecules, including the Z sex chromosome. The mitochondrial genome is 16.97 kb long.

 The genomic data are publicly accessible through the NCBI database (GCA_963854805.1), ensuring transparency and availability for further research. Additionally, the curation pipeline is available on GitHub, providing an open-source resource for validation and improvement. The study's protocols are appropriate, and the methodologies employed are technically sound. The detailed description of methods and materials allows for reproducibility, facilitating further research in this area. Moreover, the datasets are clearly presented in a usable and accessible format, enhancing their utility for the scientific community.

Are sufficient details of methods and materials provided to allow replication by others?

Yes

Is the rationale for creating the dataset(s) clearly described?

Yes

Are the datasets clearly presented in a useable and accessible format?

Yes

Are the protocols appropriate and is the work technically sound?

Yes

Reviewer Expertise:

genetics and genomics

I confirm that I have read this submission and believe that I have an appropriate level of expertise to confirm that it is of an acceptable scientific standard.

# Reviewer report: Reviewer response for version 1

**DOI:** 10.21956/wellcomeopenres.26209.r119961

**Published:** 2025-03-10

**Authors:** Laurence Despres

**Affiliations:** 1 Universite Savoie Mont Blanc, Auvergne-Rhône-Alpes, France

The authors report on the chromosome-level genome assembly of an individual male specimen of *Plagodis dolabraria* (the Scorched Wing; Geometridae). The initial identification by morphology of the two collected specimens was verified by an additional DNA barcoding process. Given the distinctiveness of the Scorched Wing, this additional sequencing step could have been avoided. I have some reserves regarding the systematic inclusion of a barcode process in the standardized DToL procedures when it is not necessary; my main concern is keeping science as sober as possible.

 The genome sequence is 939 megabases in span. Most of the assembly is scaffolded into 31 chromosomal pseudomolecules, including the Z sex chromosome. The Z chromosome was assigned by synteny to *Agriphila geniculea* (GCA_950108535.1). The assembly was performed using appropriate methods : Pac Bio HiFi long reads (90-fold coverage) and Hi-C data to refine scaffolding, manual curation of the final assembly, and contamination check, following the analysis pipeline and high standard of the Darwin Tree of Life Project for results presentation.

 The mitogenome was also assembled. The quality of the assembly was further assessed by the proportion of complete BUSCO genes recovered, which is very high (>98% of Lepidoptera database BUSCO genes lepidoptera_odb10 set). Annotation was apparently not performed, although protein-coding predictions would be a useful addition to this high quality genome.

 This high quality genome assembly will provide a reference genome for further genomic studies on this geometrid moth.

Are sufficient details of methods and materials provided to allow replication by others?

Yes

Is the rationale for creating the dataset(s) clearly described?

Yes

Are the datasets clearly presented in a useable and accessible format?

Yes

Are the protocols appropriate and is the work technically sound?

Yes

Reviewer Expertise:

evolutionary biology

I confirm that I have read this submission and believe that I have an appropriate level of expertise to confirm that it is of an acceptable scientific standard.
